# Utilisation of Learning Analytics to Identify Students at Risk of Poor Academic Performance in Medical Schools

**DOI:** 10.7759/cureus.66278

**Published:** 2024-08-06

**Authors:** Thai Ling Wong, David Hope, Alan Jaap

**Affiliations:** 1 Medical Education, The University of Edinburgh, Edinburgh, GBR

**Keywords:** academic performance/grades, preclinical education, online medical education, learner engagement, learning analytics

## Abstract

Introduction

Identifying students at risk of failure before they experience difficulties may considerably improve their outcomes. However, identification techniques can be costly, time-intensive, and of unknown efficacy. Medical educators need accessible and cost-effective ways of identifying at-risk students. The aim of this study was to investigate the relationship between student engagement in an online classroom and academic performance given the transition of many courses from in-person to online learning.

Methods

A retrospective study was conducted on a group of 235 students from the University of Edinburgh Bachelor of Medicine and Surgery (MBChB) in Year One for eight weeks from the start of term, September 2020. Purposive sampling was used. Data were collected on total test submissions, total discussion board submissions, engagement scores, and overall exam scores. Learning analytics on discussion board engagement were collected for new medical students before they had sat any summative assessment. Tests completed, discussion board posts made, and their total engagement score were correlated with their first summative assessment scores at the end of semester one.

Results

We found a statistically significant correlation between total test submissions, total discussion board submissions, engagement scores, and overall exam scores, with small-medium effects (r = 0.281, p<0.001) (r = 0.241, p<0.001), and (r = 0.202, p<0.001). Students with more test submissions, total discussion board submissions, and total engagement had a higher overall exam score. There was a statistically significant moderate correlation between total submissions and overall exam scores (r = 0.324, p<0.001).

Conclusions

Students who had a higher number of submissions were more likely to perform better on assessments. Early engagement correlates with performance. Learning analytics can help identify student underperformance before they undertake any assessment, and this can be done very inexpensively and with minimal staff resources if properly planned.

## Introduction

Online learning has become increasingly popular amongst universities, especially after the COVID-19 pandemic. The National Centre for Education Statistics (NCES) reported that approximately 10 million students attended higher-education courses online, which was projected to increase by 20.5% between 2022 and 2030 [1]. Online learning platforms allow a more detailed analysis of individual learning patterns as compared to traditional in-person teaching.

Learning analytics provide important data on learners’ engagement online and could potentially play a crucial role in improving academic performance. Newmann defined engagement as “the student’s psychological investment in an effort directed towards learning, understanding, or mastering the knowledge, skills, or crafts that academic work is intended to promote” [2]. While there is extensive research on whether student engagement improves academic achievement, the findings are mixed, with a large number of studies showing a positive correlation while others found minimal or no correlation between engagement level and academic grades [3-7]. A better understanding of the effects of engagement through online learning management can help educators design effective online learning activities and materials and introduce new policies to improve student engagement and their overall performance.

In 2019, tutorials for medical students in Edinburgh were delivered virtually as a consequence of the COVID-19 pandemic. In addition, university admissions for the term 2020/2021 based on A-Level predicted grades [8] led to some uncertainty about the students’ actual academic ability. These events, coupled with the implementation of learning analytics for online classrooms, highlighted the importance of effective use of learning analytics to potentially improve student retention and academic achievements. Furthermore, preclinical grades are predictors of academic performance in clinical years [9]. This is why it is imperative to be able to utilise learning analytics effectively by better understanding the relationship between student engagement and learning outcomes in order to help identify students at risk of poor outcomes to trigger immediate intervention in preclinical years.

To our knowledge, this is the first study in Scotland to utilise learning analytics to examine the relationship between online engagement and academic performance amongst preclinical students. The aim of this study was to investigate the relationship between student engagement in an online classroom and academic performance with the utilisation of learning analytics, given the transition of many courses from in-person to online learning. 

## Materials and methods

Data collection

A retrospective study was conducted on 235 students from the University of Edinburgh Bachelor of Medicine and Surgery (MBChB) in Year One. Purposive sampling was used. Data were collected over eight weeks at the start of the course, from September 2020.

The study was conducted on all Year One students for the academic year 2020/2021 to allow early identification of students at risk of poor academic performance for early intervention. This was due to the uncertainty of students’ actual academic performance due to the change of admissions criteria, which was based on predicted A-level grades. 

The topics covered in Semester One of Year One MBChB included physiology, anatomy, clinical pharmacology, knowledge of clinical practice (KCP), social and ethical aspects of medicine (SEAM), and research and evidence-based medicine (REBM).

All tutorials, quizzes, and forum discussions were delivered online on Blackboard Learn and Blackboard Collaborate Ultra (Anthology Inc., Boca Raton, FL). Blackboard Analytics for Learn (Anthology Inc.) was used to collect data for the study [10]. Data collected included the total test submissions, total discussion board submissions, total submissions, engagement scores, and total exam scores.

Total test submissions 

During each week of the Year One MBChB Programme, weekly formative quizzes were made available to encourage active learning and revision. Unlimited reviews of the formative assessments were made available for revision throughout Semester One. There was no time limit set for the formative assessments. The total test submissions were calculated by the total number of submissions of formative quizzes attempted by each student.

Total discussion board submissions

Both students and instructors were provided with access to the Blackboard Learn Discussion Boards. Discussion boards were regularly monitored and facilitated by the respective course leads. Students were able to create posts to engage in in-depth discussions with peers and instructors regarding relevant topics. The total discussion board submissions were measured by the total number of posts created on Blackboard Learn by the students. 

Total submissions

We combined the total number of submissions made for formative tests and discussion board activities to measure the total number of submissions for each student. 

Engagement score

The engagement level of students was monitored throughout Semester One. The engagement of each student was monitored during each tutorial, based on the duration of time spent on each course and online course materials, attendance, and frequency of access to courses.

Total exam score

A summative assessment was conducted at the end of Semester One. All assessments were delivered remotely. The summative assessment tested the knowledge of the content covered during Semester One. The scores were calculated out of 90 marks, and feedback was made available to students after the assessment. 

Eligibility 

Participants of the study were newly enrolled Year One students of the MBChB Programme at the University of Edinburgh during the term 2020/2021.

Ethics

Ethical approval was obtained from the University of Edinburgh Medical Education Ethics Committee (reference number: 2020/33). Students involved in the study consented to the routine evaluation of their data. All data included in this study were anonymised before data analysis was performed. Researchers involved were not able to identify individual medical students at any point of the study. 

Data analysis 

The data collected were analysed using IBM SPSS Statistics for Windows, version 27.0 (IBM Corp., Armonk, NY). Descriptive statistics used were mean and standard deviation. A Pearson’s correlation test was used to find out the correlation between the variables and students’ performance. A scatter plot was drawn to determine the relationship between the variables and the total overall score. 

## Results

**Figure 1:**
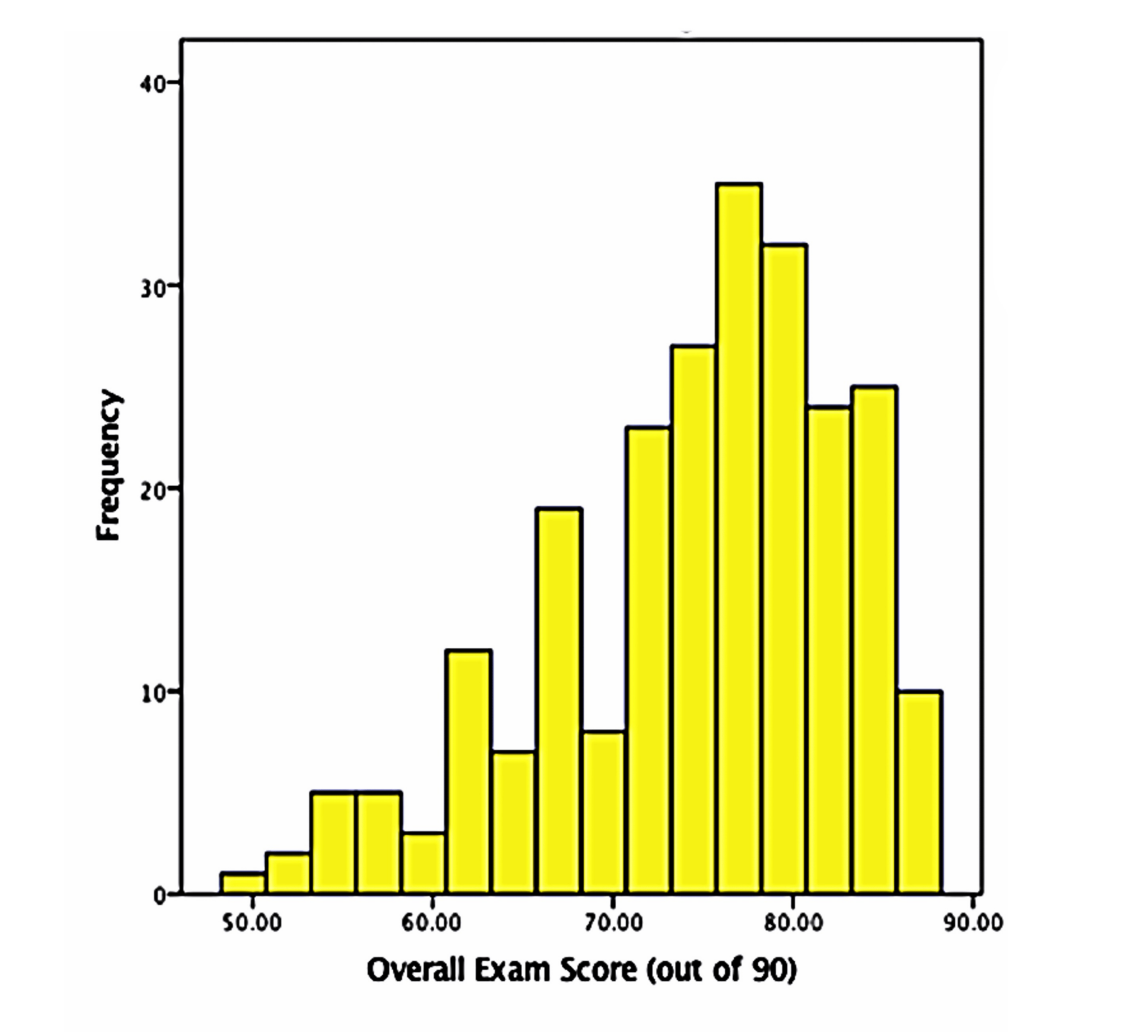
Histogram of the overall exam score obtained from the semester one summative assessment

Overall, a total of 235 students in Year One of the University of Edinburgh MBChB Programme were included in this study. The distribution of the overall exam score calculated using the summative assessment conducted at the end of semester one is shown in the histogram in Figure 1. The histogram shows a left-skewed distribution of the overall exam score out of 90 marks. Most students obtained a score between 70 and 80 marks. The highest score obtained was 88 out of 90 marks, while the lowest score was 49.5 out of 90 marks. 

The characteristics of the total number of test and discussion board submissions, engagement score, and overall exam score are shown in Table 1. A total of 235 submissions were completed for formative tests and discussion board activities. The mean submissions for formative quizzes were 4.89 ± 2.00 submissions per student. Meanwhile, the mean submissions for the Blackboard Discussion Board activities were only 1.85 ± 1.08 submissions per student. The mean submission for the overall submissions (sum of test and discussion board submissions) was 6.73 ± 2.55 submissions per student. The mean engagement score was 105.32 ± 1.55. A total of 233 students taking the Year One exam subsequently completed the final compulsory summative assessment. The mean overall exam score was 74.40 ± 8.15 marks (out of 90 marks).

**Table 1:** Characteristics of total submissions, total test submissions, total board discussions, engagement scores, and overall exam scores of all included students

Variables	Total submissions		Engagement score	Overall exam score (out of 90)
	Total test submissions	Total discussion board submissions		
Total, n	235	235	236	233
Mean ± SD	4.89 ± 2.00	1.85 ± 1.08	105.32 ± 1.55	74.40 ± 8.15
Total Mean ± SD	6.73 ± 2.55			

**Figure 2:**
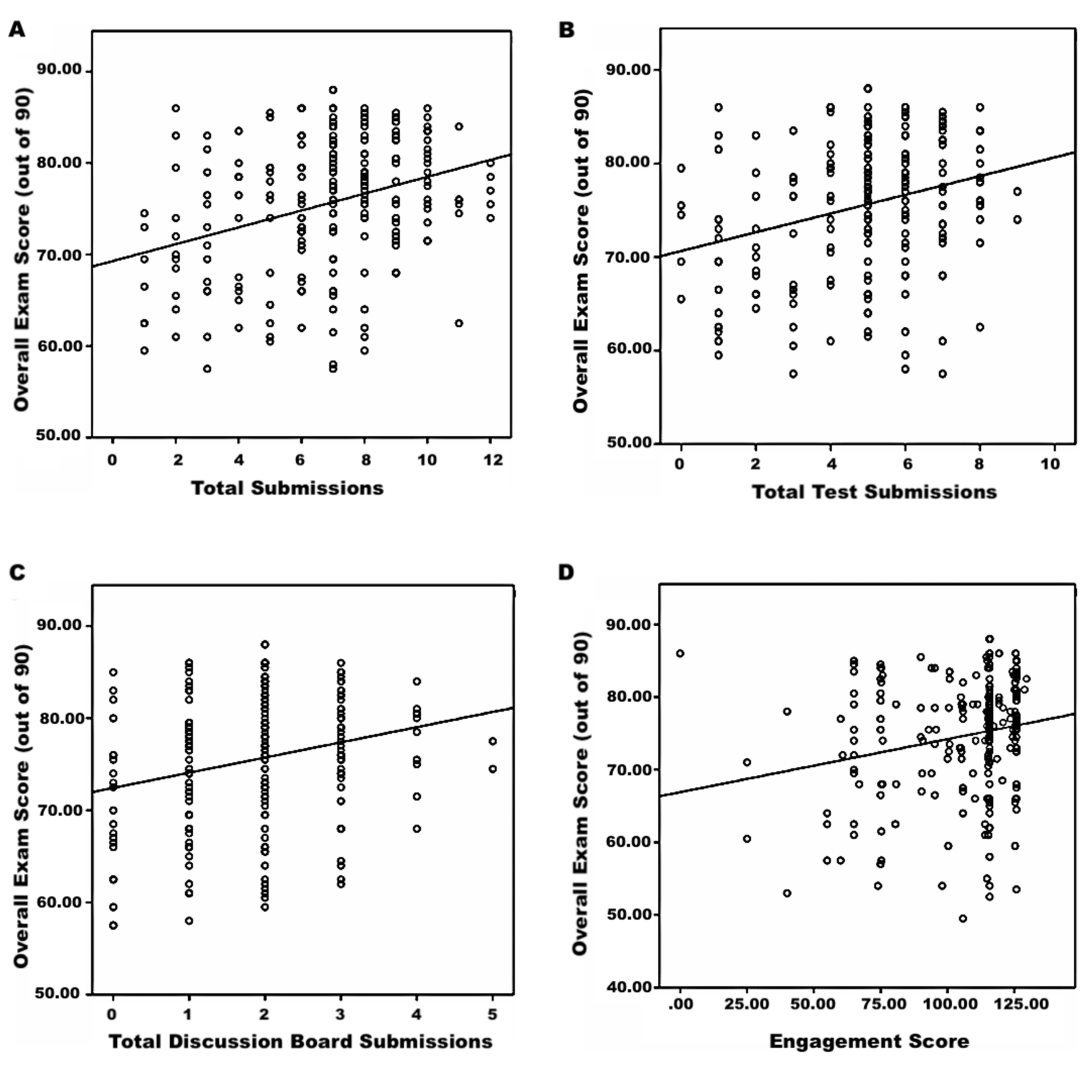
Scatter plot showing the relationship between the total submissions (a), total test submissions (b), total discussion board submissions (c), and engagement score (D) with the overall exam score

The relationship between the total number of submissions, test submissions, discussion board submissions, and engagement with the overall exam score is demonstrated in Figure 2. The results of the Pearson correlation show a significant positive correlation between total test submissions, total discussion board submissions, and overall exam score (r = 0.281, p <0.001), (r = 0.241, p <0.001), with small effects. Students who had completed a higher number of tests or total discussion board submissions were likely to achieve a better overall exam score.

Similarly, there was a significant positive correlation between the engagement score and overall exam score (r = 0.202, p <0.001), with small effects. Those with better engagement scores obtained by active participation in classes, having better engagement with online course content, and spending more time on online course materials were likely to perform well in their summative assessments. 

There was a moderate correlation between total number of submissions and overall exam score (r = 0.324, p <0.001). Students who completed more formative quizzes and were more involved in the Blackboard Discussion Board activities were more likely to achieve a better academic performance in their summative assessments as compared to other students.

## Discussion

The significant, weak positive correlation between student engagement and overall exam score could be explained by several factors. First, increased teacher-student interaction and interaction between peers could have improved student performance through the exchange of feedback, reduced social isolation, and an increased level of affirmation. The online learning environment encourages the exchange of feedback between learners and tutors and amongst learners themselves through collaborative discussion forums and formative test submissions. Zimbardi et al. demonstrated that time spent accessing tutors’ feedback was significantly associated with improvement of academic outcomes [11]. Similarly, peer feedback was also shown to effectively improve academic achievement by promoting interaction, engaging students in their own process of learning, and encouraging learners to better understand an examiner's perspective [12]. In addition, affirmation can be delivered through positive feedback shown to effectively improve academic performance [13]. Besides that, increased interaction in online classrooms could have contributed to overcoming social isolation. Studies have found that social isolation was more likely to negatively affect educational outcomes [14, 15]. Our study was conducted during a period when medical students in Edinburgh were legally required to self-isolate to mitigate the spread of COVID-19. In-person on-campus sports and social activities were suspended at the time. The increased interaction among peers and with tutors could have helped reduce the feeling of isolation, which in turn, positively affected educational outcomes.

Second, engagement with learning activities could be associated with the affective elements of the learning process, such as personality, motivation, effort, and self-confidence. Learners demonstrating more motivation and dedication tend to do well academically and are more likely to spend more time engaging with learning activities and therefore perform better [16]. Ditta et al.’s study with the aim to address low motivation levels found that exposure to new topics motivated students to broaden their knowledge [17]. Hence, although better academic performance could be a result of higher motivation levels, effective techniques and teaching models can also be introduced by educators to, in turn, increase the learners’ motivation. In addition, the utilisation of online discussion boards could help learners overcome social anxiety and low levels of self-confidence. For example, anonymous discussion boards within the learning management systems would encourage learners to ask questions without the fear of being identifiable. An online survey showed that students were significantly more likely to post when anonymous posting was enabled [18]. Therefore, affective elements of learning such as motivation, personality, and self-confidence could affect the learning outcome and should be considered when designing online teaching activities to improve engagement levels and academic performance. 

Third, the incorporation of practice and repetition could positively influence academic performance. Students more engaged with online learning are more likely to have repeated encounters with learning materials to facilitate their participation in online discussions, completion of formative quizzes, and review of feedback obtained. Extensive research has demonstrated spaced practice and repetition as an effective way of improving learning and educational outcomes [19]. The availability of online resources enables spaced-out repetition and familiarisation of learning materials. Furthermore, knowledge retention was associated with a deep level of understanding of a topic [20]. Recorded tutorials enable learners to revisit lectures, repeat quizzes, or exercises and revise at their own pace. This allows better comprehension of a topic and improves a learner's depth of learning.

Challenges of improving engagement in online learning environments

The challenges in implementing change to improve learners’ engagement in an online environment are manifold. Socially deprived areas with a lack of access to stable internet could impede online participation and student engagement. Several institutions have attempted to address this issue by providing financial support and scholarships to students facing barriers to accessing online education. Whilst these efforts are commendable, sustainable solutions are required to ensure equal access to education. Furthermore, the online learning environment has a negative impact on students with learning disabilities, further widening the learning gap that was already present before the introduction of online learning [21]. Personalised learning environments should be introduced to account for students of different backgrounds and learning needs. Moreover, online learning can have a long-term impact on the psychological well-being of learners [22]. The benefits of a balanced mix of online and in-person teaching should be further explored to ensure effective learning.

Limitations

We acknowledge that this study has some limitations. The rise in online educational projects, such as online tutorials on YouTube and Osmosis, also meant that we were unable to identify students who were disengaged with course activities but actively revising other online materials readily available to medical students. Besides that, this study was based on a correlation design, which does not infer a cause-and-effect relationship between engagement and academic performance. We also assumed that students present at tutorials were actively participating in sessions. Furthermore, our analytics did not record active engagement during live sessions, such as the number of questions asked or polls answered. In addition, with the shift to hybrid teaching and learning in higher education, further studies would be required to better identify the effects of engagement and academic performances in hybrid classrooms. 

## Conclusions

In conclusion, the level of engagement and submissions made by students directly correlates with the overall performance of students in exams. The use of learning analytics in identifying students at risk of failure provides an opportunity for educators to intervene before students experience difficulties. While online learning has its own benefits, it is important to take into consideration the advantages of face-to-face learning and incorporate hybrid teaching where appropriate. Strategies such as incorporation of active learning, providing feedback to students, and activities encouraging active participation should be introduced for students identified to be at risk of poor academic performance. 
